# *ELOC*(*TCEB1*)-mutated renal cell carcinoma: a case report and clinicopathological analysis

**DOI:** 10.3389/fonc.2025.1703364

**Published:** 2025-11-20

**Authors:** Yin Zhu, Mengchao Fang, Shuo Wang

**Affiliations:** 1 Department of Pathology, Ningbo Yinzhou No. 2 Hospital, Ningbo, China; 2 Hangzhou Cancer Institution, Hangzhou Cancer Hospital, Hangzhou, China

**Keywords:** RCC (renal cell carcinoma), ELOC(*TCEB1*), case report, clinicopathological, immunohistochemistry

## Abstract

*ELOC* (also referred to as *TCEB1*)-mutated renal cell carcinoma (*ELOC*-mutated RCC) is a rare, molecularly defined RCC newly incorporated into the 2022 5th Edition WHO Classification of Tumours of the Urinary and Male Genital Organs. It exhibits a broad histomorphological spectrum with overlapping features with clear cell RCC, posing diagnostic challenges and potential for misdiagnosis if not considered. This report presents a case initially diagnosed as CK7-positive RCC with fibromyomatous stroma (RCC-FMS). Next-generation sequencing (NGS) revealed a *ELOC* p.Y79C gene mutation in tumor cells, leading to the definitive diagnosis of *ELOC*-mutated RCC. Enhancing awareness of this rare tumor is crucial for improving diagnostic accuracy.

## Introduction

The 2022 WHO classification introduced several new molecularly defined categories of RCC, including those with *TFE3* rearrangements, *TFEB* rearrangements, *TFEB* amplification, *FH* deficiency, *SDH* deficiency, *ALK* rearrangements, *ELOC* mutations and *SMARCB1* (*INI1*) deficiency RCC (1, 2). Characterized by its distinctive features and immunophenotypic profile, *ELOC*-mutated RCC is one such newly recognized entity (3). Approximately 70% of clear cell RCCs are characterized by biallelic inactivation of the von Hippel-Lindau (*VHL*) gene on chromosome 3p, caused by 3p deletion, *VHL* mutation, and/or *VHL* promoter hypermethylation. Cases of RCC with smooth muscle stroma were first reported by Canzonieri et al. in 1993, in a patient who also had an angiomyolipoma (4). In 2013, Sato et al. first identified a subset of RCCs lacking 3p deletion (monosomy) and/or VHL mutation but harboring characteristic hotspot mutations in *ELOC* on chromosome 8q, along with loss of heterozygosity at 8q, defining *ELOC*-mutated RCC (5). Although subsequent cases have been reported, it remains a rare subtype with only several dozen cases described in the literature (6–10).

## Case presentation

**Figure 1:**
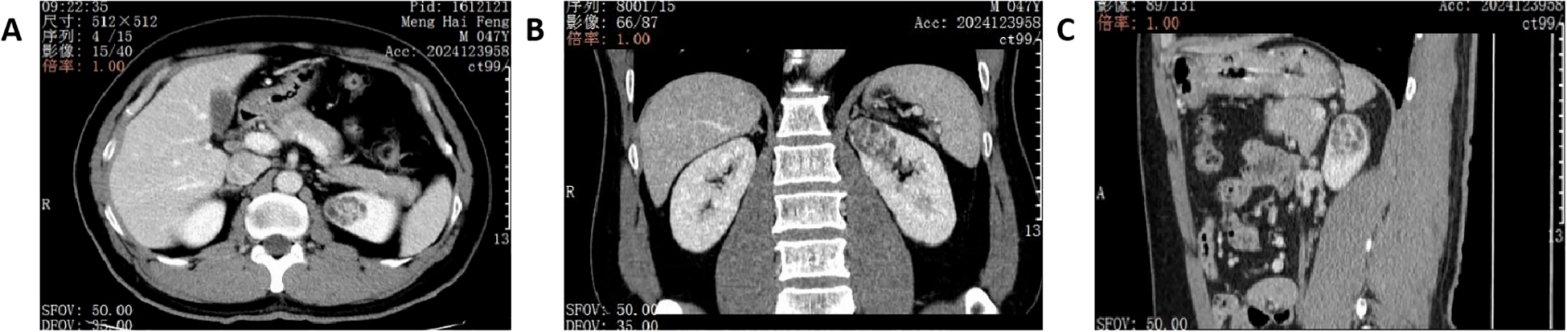
Renal CT images on admission. **(A)** axial plane of CT image. **(B)** coronal plane of CT image. **(C)** sagittal plane of CT image.

A 47-year-old male was admitted in June 2024. During a routine physical examination at a local hospital, a full abdominal magnetic resonance imaging (MRI) revealed a left renal tumor. The patient’s medical history includes a 5-year history of hypertension managed with amlodipine besylate, a 10-year history of chronic hepatitis B virus (HBV) infection, a 20-year smoking history, elevated transaminase levels, and a 10-year history of chronic gastritis. The patient denied any family history of renal malignancies. In our hospital, abdominal computed tomography (CT) revealed a space-occupying lesion in the left kidney, highly suggestive of RCC. The lesion was closely associated with branches of the left upper segmental renal artery. A round, low-density shadow approximately 33*25 mm in size was observed in the parenchyma of the left upper pole. It showed marked heterogeneous enhancement on contrast-enhanced scans, more pronounced in the cortical phase and slightly higher than the normal renal cortex, appearing as a relative low-density area in the excretory phase (**Figure 1**). The R.E.N.A.L. nephrometry score for the lesion was 7 points, derived from the following parameters: (R) tumor radius, defined by the maximum axial diameter of 41 mm, which confers 2 points; (E) exophytic component, with more than 50 % of the mass extending beyond the renal contour, yielding 1 point; (N) nearness to the collecting system or renal sinus, measured at less than 4 mm, resulting in 3 points; (A) anterior location; and (L) position relative to the polar lines of the left kidney, which adds 1 point. The tumor exhibited a well−defined margin without any evidence of invasion, and the adjacent renal calyces retained their normal architecture, showing no discernible structural alteration. A left partial nephrectomy was performed two days later.

### Pathological examination

**Figure 2:**
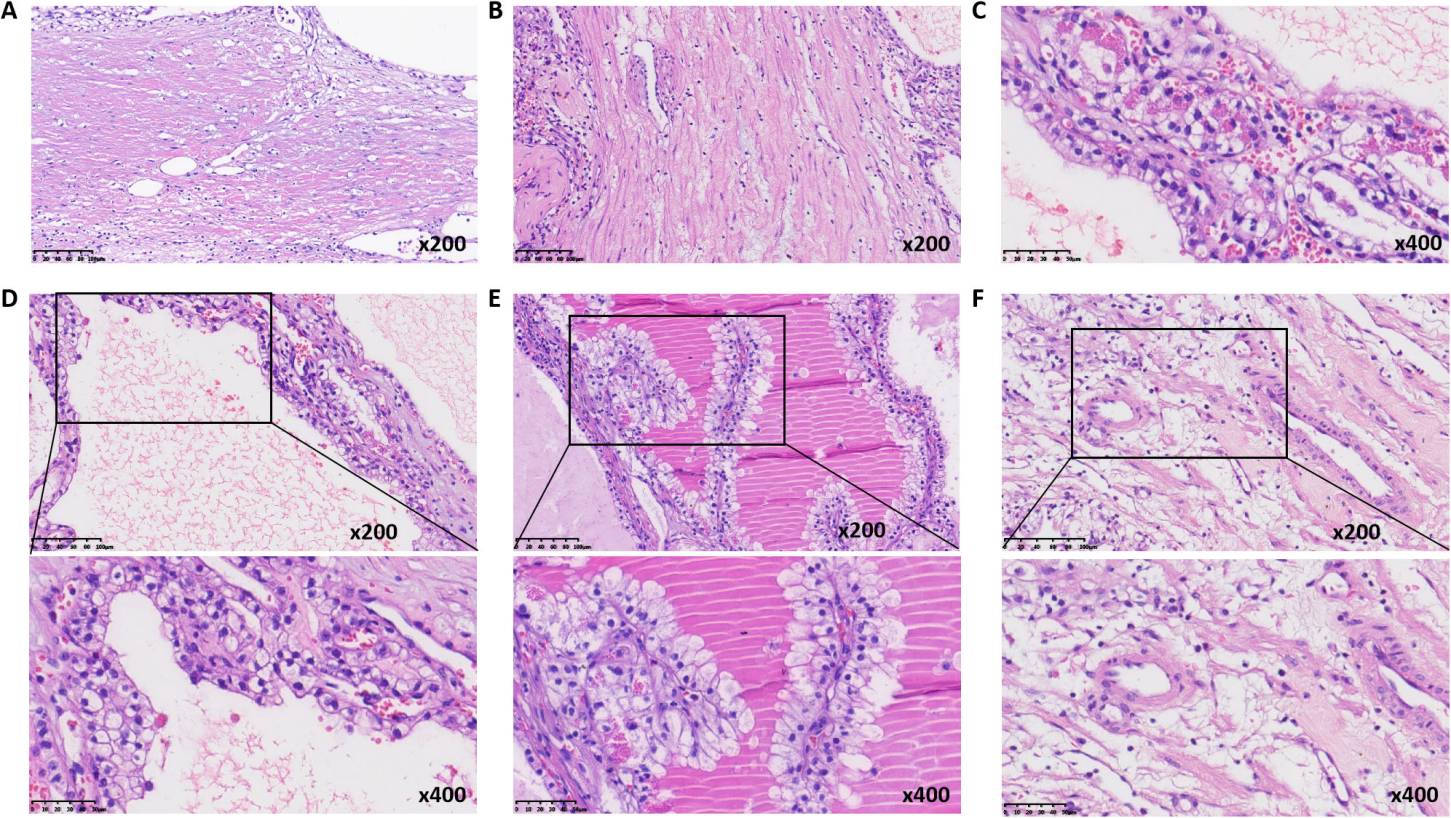
Histologic morphology of ELOC-mutated RRC. **(A)** thick fibrous capsule surrounding the tumor (medium magnification). **(B)** fibromuscular septa (medium magnification). **(C)** cytoplasmic eosinophilic granules (high magnification). **(D)** tubular and cystic structures (high magnification). **(E)** papillary structures (high magnification). **(F)** muscular blood vessel (high magnification).

**Figure 3:**
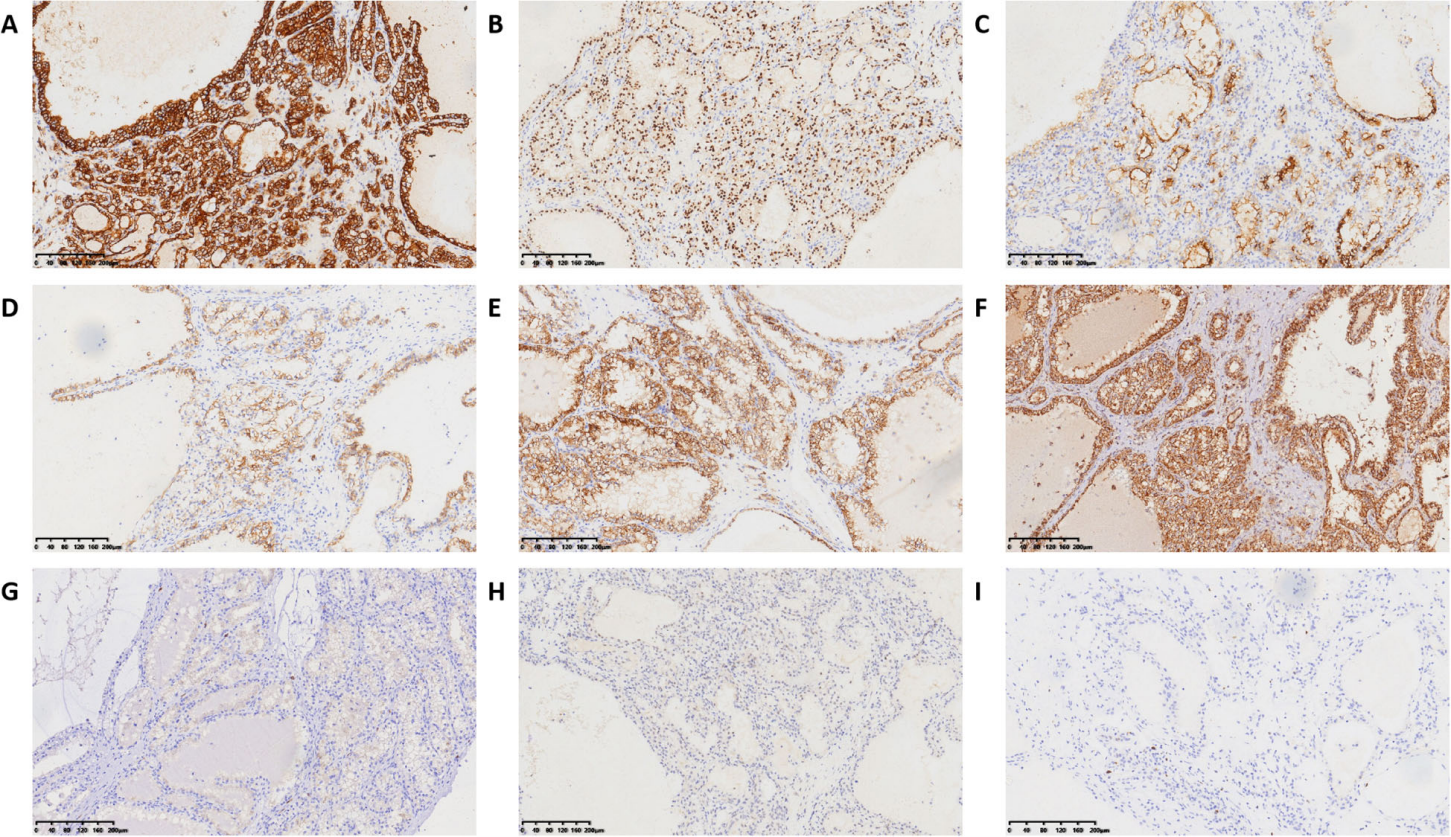
Immunohistochemical results of ELOC-mutated RRC. **(A)** positive for CA IX. **(B)** positive for PAX8. **(C)** positive for CD10. **(D)** positive for CK7. **(E)** positive for AMACR. **(F)** no FH-deficient. **(G)** positive for CD117 in mast cells. **(H)** negative for TFE3. **(I)** positive for Ki-67 in approximately 5%.

**Figure 4:**
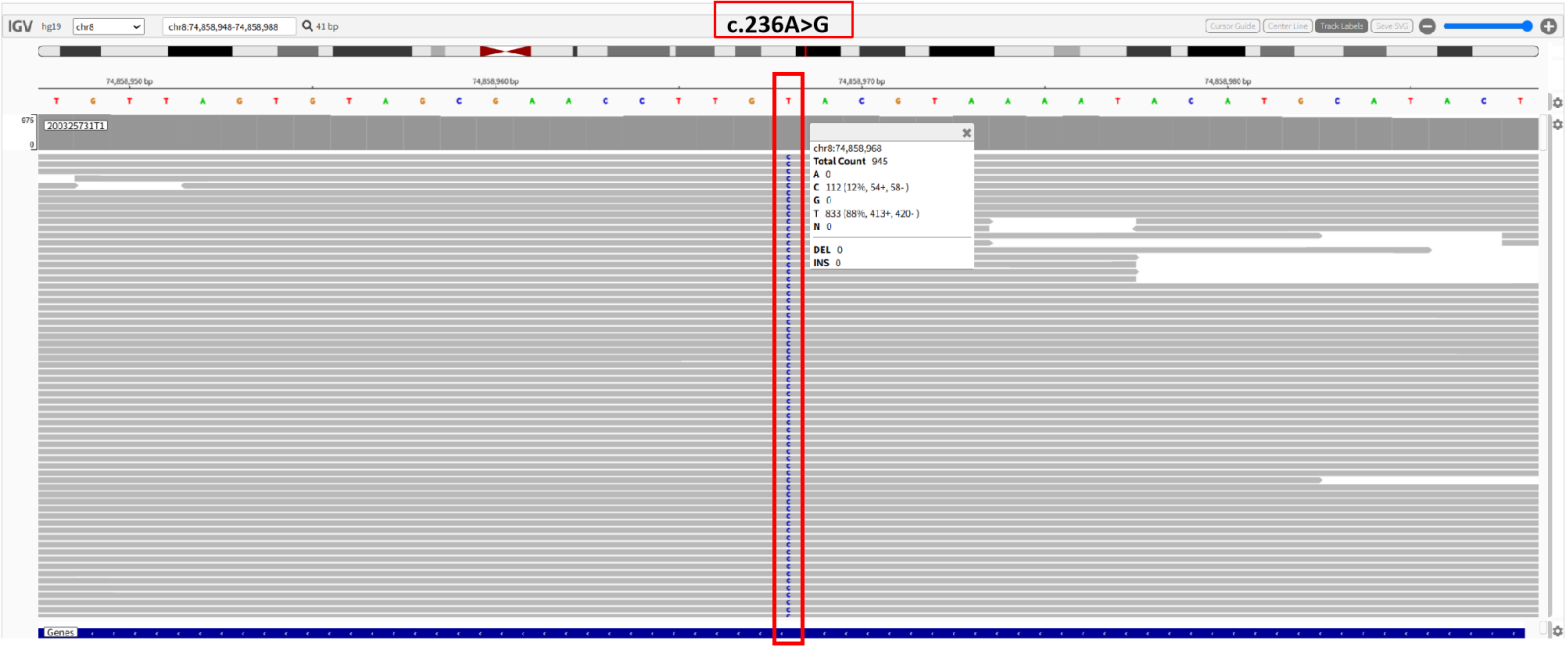
Sequencing analysis of the c.236A>G mutation.

The partial nephrectomy specimen measured 3.5 cm*3 cm*2.2 cm. The tumor itself measured 2.9 cm*2.9 cm*2.4 cm. The cut surface was variegated and encapsulated. Microscopically, the tumor was well-circumscribed with a thick fibrous capsule and exhibited pushing borders. Tumor cells were arranged predominantly in cystic and acinar patterns, with focal nested and papillary architectures. Fibrosmooth muscle-like stroma interspersed among the tumor cells. Tumor cells had abundant clear cytoplasm with distinct cell borders. Nuclei were round or oval, with fine chromatin and inconspicuous nucleoli. Focal eosinophilic granules were observed within the cytoplasm (**Figure 2**). The morphology was suggestive of a low-grade RCC. Immunohistochemical staining showed tumor cells were positive for PAX8, CA9, CK7, and AMACR (also referred to as P504S), partially positive for CD10, and negative for TFE3 and CD117. The positive for Ki-67 was approximately 5% (**Figure 3**). The co-expression of CA9 and CK7, combined with the fibrosmooth muscle stroma, prompted consideration of other molecularly defined renal carcinomas, such as those with *ELOC* mutations or TSC/mTOR pathway alterations. Given the scarcity of reported cases, an NGS panel in tumor cells covering 808 genes was performed to exclude common tumors like clear cell RCC. NGS was performed using the MGI platform at an average depth of 2389X, revealing a variant in the *ELOC* gene with an allele frequency of 11.91%. Copy number variation analysis showed no significant abnormalities. Results identified a point mutation (non-synonymous) in *ELOC* p.Y79C, specifically a c.236A>G variant in exon 6 leading to an p.Y79C amino acid change (**Figure 4**). We performed loss of heterozygosity (LOH) analysis targeting the 8q21 region on chromosome 8. The testing panel covered 808 tumor-associated genes, interrogating point mutations, insertions/deletions, copy number alterations, and gene fusions. While 31 somatic mutations were identified, LOH at 8q21 was not detected. No mutations were detected in *TP53*, *BAP1*, *BRAF*, *RET*, *SETD2*, *VHL*, *mTOR* or *TSC* genes. Specifically, results revealed the absence of single nucleotide variant (SNV) or insertion/deletion (Indel) mutations in the *VHL* gene, which excluded VHL inactivation.

### Pathological diagnosis

(Left Kidney) *ELOC*-mutated RCC. The pathological assessment confirmed an ISUP Grade 2 renal cell carcinoma, pathological stage pT1aN0M0. The tumor showed absence of necrosis and no sarcomatoid or rhabdoid differentiation. All surgical margins were negative.

### Surgery

The patient underwent a laparoscopic left partial nephrectomy. The renal artery was clamped for 15 minutes, with an estimated intraoperative blood loss of 50 mL. The procedure was well-tolerated without complications, and final pathology confirmed negative surgical margins.

### Follow-up

The patient had an unremarkable postoperative recovery. While initial imaging indicated minor perirenal effusion, a subsequent CT examination in June 2025 (Six months after partial nephrectomy) confirmed its complete resolution and revealed no indications of local recurrence or distant metastasis.

## Discussion

The *ELOC* gene, located at 8q21, encodes Elongin C, a subunit of the transcription factor B (SIII) complex involved in RNA elongation during transcription. It is also a crucial component of the VHL complex within the E3 ubiquitin ligase complex. Under normoxic conditions, this complex targets hypoxia-inducible factor HIF-1α for ubiquitination and degradation, preventing a hypoxic response. Mutations in *ELOC* lead to loss of Elongin C function, impairing the binding site for the VHL complex and consequently preventing ubiquitination and degradation of HIF. This results in accumulation and overexpression of HIF-1α, which ultimately activates the transcription of numerous oncogenes, driving tumorigenesis (11). Past research focused predominantly on clear cell RCC with VHL inactivation. Avgi Andreou et al. investigated the role of ELOC in VHL-independent renal tumors, searching for germline and somatic *ELOC* mutations (9). They found no evidence of pathogenic germline *ELOC* variants in 91 individuals with a VHL-like phenotype. However, among 1,336 RCC samples from the 100,000 Genomes Project, they identified 8 RCCs with somatic pathogenic ELOC mutations, showing 8q loss and negative for VHL mutation. Aashil A. Batavia et al. identified 18 RCCs with *ELOC* deletion among 123 VHL-wildtype renal tumors, three of which harbored *ELOC* mutations leading to biallelic inactivation, indicating *ELOC* mutations can occur in both VHL-inactivated and VHL-wildtype RCCs (12). Our case had a *ELOC* point mutation with wildtype VHL protein and no detected *VHL* mutation. Future attention could be paid to RCCs with concurrent *VHL* and *ELOC* mutations.

Due to crosstalk between the TSC1/TSC2, mTOR, and ELOC pathways, and their overlapping histological and immunohistochemical features, some authors have grouped these tumors under the term “RCC with leiomyomatous stroma”. Some studies suggest that sporadic RCCs with *TSC1*/*TSC2*, *mTOR* and/or *ELOC* mutations belong to the same family of renal tumors as RCCs associated with hereditary tuberous sclerosis complex (TSC) (10). TSC, or Bourneville disease, named after Bourneville who discovered cerebral sclerotic nodules in 1880, is a neurocutaneous syndrome characterized by abnormal cell differentiation and proliferation, neuronal migration defects, and hamartomatous growths like angiomyolipomas in various organs (13). Its classic clinical triad includes epilepsy, intellectual disability, and facial angiofibromas. Our patient did not exhibit these features, and no *TSC* gene mutations were detected. However, this association warrants attention in future cases.

Based on limited literature (6–10), ELOC-mutated tumors typically occur in middle-aged and elderly adults (age range 31–78 years), with a male predominance. Tumors are usually solitary, ranging from 1.0 to 3.8 cm in diameter, and staged between pT1 and pT3a. Grossly, they are located in the renal cortex, well-circumscribed, solid nodules with a tan to brown cut surface. Microscopically, they often have a thick fibrous capsule and grow in nests, tubules, and papillae. Fibromyomatous stroma commonly infiltrates between tumor cells, creating a lobulated, nodular appearance at low power. Tumor cells have clear cytoplasm. Immunohistochemically, they are positive for CA9, CD10, PAX8, and CK7. CA9 typically shows diffuse “box-like” staining. AMACR shows variable cytoplasmic intensity, from focal weak to diffuse strong positivity (8). The fibromuscular stroma is often positive for smooth muscle actin and desmin. The morphology of *ELOC*-mutated RCC overlaps with other RCC subtypes, particularly clear cell RCC (ccRCC) and clear cell papillary renal cell tumor (CCPRCT), leading to potential misdiagnosis. Unlike ccRCC, *ELOC*-mutated RCC expresses CK7 and lacks VHL gene inactivation. Unlike CCPRCT or related renal angiomyoadenomatous tumors, *ELOC-*mutated RCC lacks prominent subnuclear vacuoles, and CCPRCT typically shows a “cup-shaped” CA9 staining pattern. The reported case showed mostly cystic and acinar patterns with focal nested and papillary arrangements, interspersed with fibromyomatous stroma. Tumor cells had abundant clear cytoplasm, distinct borders, round/oval nuclei with fine chromatin and inconspicuous nucleoli. Focal eosinophilic cytoplasmic granules were noted. Immunohistochemistry was positive for CA9, CD10, CK7 and PAX8. CD117 highlighted scattered mast cells within the tumor. FH expression was retained, and TFE-3 was negative. The histology and immunohistochemical profile are consistent with previous reports. The prominent eosinophilic granules in some tumor cells caught our attention, a morphological feature also observed in a previously reported case (14). *ELOC* p.Y79C mutation appears to represent a hot spot for *ELOC* mutation. Wang et al. identified the p.Y79C and p.E92K mutation as potential hotspot in multiple cases (15). Moreover, a cohort study by Wu et al. reported that 9 out of 15 *ELOC*-mutated RCC cases harbored the novel p.E92K mutation (16). Observing this feature in subsequent cases may help determine if it is associated with *ELOC* mutation.

*ELOC*-mutated RCC generally exhibits indolent biological behavior with a favorable prognosis, though approximately 10% of cases may metastasize (11). Literature suggests metastasis may be associated with higher nuclear grade and stage; high-grade tumors might harbor additional mutations in genes like *TERT*, *PMS2* or *NF1* alongside the *ELOC* mutation (6). Identifying *ELOC*-mutated RCC helps define a subset of RCC with low metastatic risk. Accurate diagnosis can prevent unnecessary adjuvant therapy for these indolent, completely excised tumors and avoids confounding clinical trial results. In our case, a 5-month postoperative CT showed no recurrence or metastasis; continued follow-up is ongoing.

While molecularly defined cancers are attractive for targeted therapy, the limited availability of drugs for specific genomic alterations poses challenges for treating molecularly defined RCCs. Many molecular subtypes respond poorly to classic ccRCC therapies, making the development of specific inhibitors highly anticipated. It is hoped that in the near future, each molecularly defined subtype will have its own tailored treatment approach.

### Differential diagnosis

We summarize the characteristics of ELOC-mutated RCC, clear cell papillary renal cell tumor, and renal cell carcinoma with fibromyomatous stroma (**Table 1**).

**Table 1:** The characteristics of ELOC-mutated RCC, clear cell papillary renal cell tumor, and renal cell carcinoma with fibromyomatous stroma.

Tumor type	ELOC-Mutated RCC	Clear cell papillary renal cell tumor	Renal cell carcinoma with fibromyomatous stroma
Histomorphology	Solid nests, acinar or tubular structures; tumor cells with clear or pale cytoplasm, small and hyperchromatic nuclei, occasionally scattered large bizarre nuclei. Fibromyomatous stroma may extend into tumor cells, separating tumor into lobules with a nodular appearance under low magnification.	Branching papillary or tubulopapillary structures; clear cytoplasm, linear nuclear arrangement (away from basement membrane, toward the luminal surface), no necrosis.	Infiltrative growth; epithelial components mixed with dense spindle cell stroma. Epithelial components may be tubular or trabecular, with eosinophilic or clear cytoplasm. Stroma is smooth muscle-like (fibromyomatous).
Immunohistochemistry	CA9 (diffuse positive, box-like) P504s (positive) CK7 (positive or negative)	CA9 (diffuse strong positive, cup-like) CK7 (diffuse strong positive) GATA3 (positive) P504s (negative or weak positive)	PAX8 (positive in tumor epithelial cells) SMA or Desmin (positive in tumor stroma) CK7 (positive or negative)
Molecular Features	Somatic mutation of ELOC gene	No specific driver mutations.	No specific driver mutations. May be associated with TSC/mTOR pathway abnormalities.
Clinical Features	Intermediate invasive behavior, between ccRCC and low-malignant potential tumors in diameter; metastasis may occur.	Indolent biological behavior, excellent prognosis, almost never metastasizes; curable by surgical resection.	Diverse biological behavior, ranging from indolent to aggressive; requires grading and staging for assessment.

### Limitations

This study has limitations that merit future investigation. Despite the good general condition observed in this single case, the inherent limitations of a case report preclude generalizable conclusions about patient outcomes, underscoring the need for larger studies. Furthermore, the relatively short follow-up duration of six months warrants longer-term monitoring to evaluate the long-term prognosis.

## Conclusion

In summary, we report a case of *ELOC*-mutated RCC. This case illustrates that this subtype exhibits morphological and histopathological overlap with other entities such as ccRCC. The presence of prominent intracytoplasmic eosinophilic granules within the tumor cells may represent a characteristic feature of this subtype. The utilization of NGS enabled accurate and efficient definitive diagnosis.

## Data Availability

The original contributions presented in the study are included in the article/supplementary material. Further inquiries can be directed to the corresponding author.
